# Healthcare-Associated Infections in a Burn Treatment Unit

**DOI:** 10.1590/0100-6991e-20253882-en

**Published:** 2025-11-24

**Authors:** MARCELO MOURÃO, MARÍLIA BAENINGER, THAYSA SOBRAL ANTONELLI, DANIELA VIEIRA DA SILVA ESCUDERO, MARIA CLAUDIA STOCKLER DE ALMEIDA, ALFREDO GRAGNANI

**Affiliations:** 1 - Universidade Federal de São Paulo, Departamento de Cirurgia - Disciplina de Cirurgia Plástica - São Paulo - SP - Brasil; 2 - Hospital São Paulo, Comissão de Controle de Infecção Hospitalar - São Paulo - SP - Brasil

**Keywords:** Burns, Healthcare-Associated Infections (HAIs), Multidrug-Resistant Microorganisms, Queimaduras, Infecções Bacterianas, Transmissão de Doença Infecciosa do Profissional para o Paciente

## Abstract

**Introduction:**

Severe burns significantly weaken the immune system and disrupt the skin’s natural barrier, which increases the likelihood of healthcare-associated infections (HAIs) and raises the risk of mortality.

**Methods:**

Based on CDC criteria, this study retrospectively examines the incidence of HAIs in burn patients hospitalized at a university hospital in São Paulo, Brazil, over five years from 2018 to 2022.

**Results:**

536 patients were treated during this time, with 130 HAIs recorded in 88 individuals. The average age of the patients was 41 years, and the mean total body surface area (TBSA) affected by burns was 20.4%. The primary causes of burns were flammable liquids (39.7%), electrical injuries (25%), and scalds from heated liquids (14.8%). Burn wound infections were the most frequent HAI (51.5%), followed by bloodstream infections (13.8%), urinary tract infections (13.1%), and ventilator-associated pneumonia (10.7%). Regarding microbiological findings, 141 microorganisms were isolated, with gram-negative bacteria making up 71.6% of the total, gram-positive bacteria accounting for 21.2%, and fungi representing 7.1%. In three cases, no microorganism was identified. The mortality rate among these patients was 13,6%.

**Conclusion:**

Notably, the predominance of gram-negative bacteria in this population, responsible for more than 70% of infections, contrasts with findings from other studies. The findings highlight the importance of infection control to reduce morbidity and mortality in this vulnerable population.

## RESUMO

**Objetivo:**

As queimaduras graves prejudicam significativamente o sistema imunológico e comprometem a barreira natural de proteção da pele, aumentando a probabilidade de infecções relacionadas à assistência à saúde (IRAS) e elevando o risco de mortalidade.

**Métodos:**

Com base nos critérios do CDC, este estudo analisa retrospectivamente a incidência de IRAS em pacientes vítimas de queimaduras internados em um hospital universitário em São Paulo, Brasil, entre 2018 e 2022.

**Resultados:**

536 pacientes foram tratados durante este período, com 130 IRAS registradas em 88 indivíduos. A média de idade dos pacientes foi de 41 anos e de superfície corporal total queimada (SCQ) de 20,4%. As principais causas de queimaduras foram líquidos inflamáveis (39,7%), trauma elétrico (25%) e líquidos aquecidos (14,8%). As infecções mais frequentes foram infecções de área queimada (51,5%), infecções na corrente sanguínea (13,8%), infecções do trato urinário (13,1%) e pneumonia associada à ventilação mecânica (10,7%). Em relação aos achados microbiológicos, 141 microrganismos foram isolados, com bactérias gram-negativas representando 71,6% do total, bactérias gram-positivas 21,2% e fungos 7,1%. Em três casos, nenhum microrganismo foi identificado. A taxa de mortalidade entre esses pacientes foi de 13,6%.

**Conclusão:**

Destaca-se a predominância de bactérias gram-negativas nesta população, responsáveis pela maioria das infecções, o que contrasta com achados de outros estudos. Esses resultados ressaltam a importância do controle de infecções para reduzir a morbidade e mortalidade nesta população vulnerável.

## INTRODUCTION

Burns are among the most complex forms of trauma, often resulting in significant physical and psychological damage, and are recognized as a major public health problem. The World Health Organization (WHO) estimated that in 2019, more than 100,000 deaths were attributed to burns globally, with 90% occurring in low- and middle-income countries. Understanding the epidemiology of burns and the associated risk factors is crucial for the development of effective prevention and treatment strategies^1^ ^,^ ^2^. 

In the first few hours after burn, injuries tend to show a low level of bacterial colonization, mainly because trauma destroys the skin’s natural microbial flora. However, after about 48 hours, gram-positive bacteria from adjacent tissues begin to colonize the wound. Between the fourth and fifth day, gram-negative bacteria, often resistant to multiple antibiotics and common in hospital settings, become predominant, posing significant challenges to treatment^1^. 

Healthcare-associated infections (HAIs) are defined as infections acquired after a patient’s admission to a health care facility and may manifest during hospitalization or after discharge. In burn patients, the risk of HAI is especially high due to frequent use of invasive devices and to long hospital stay. HAIs are identified and diagnosed by the Hospital Infection Control Commission (HICC), in accordance with the infection criteria defined by the Centers for Disease Control and Prevention (CDC)^3^. 

These infections have become a major cause of morbidity and mortality among hospitalized burn patients. The disruption of the protective skin barrier creates a moist and necrotic environment in the wound, which is ideal for rapid colonization by infectious agents. This process can lead to infection, systemic invasion, and increased risk of sepsis and multiple organ failure^4^. 

The most common HAIs in this population include bloodstream infections, urinary tract infections, ventilator-associated pneumonias, and burn-area infections. Each type of infection has specific diagnostic criteria, making accurate reporting essential to production of reliable epidemiological data and development of prevention and treatment strategies^5^ ^-^ ^6^. 

## OBJECTIVE

To retrospectively analyze the incidence, topography, and microbiological agents of healthcare-associated infections (HAIs), as well as to determine the in-hospital mortality rate in a Burn Care Unit of a reference university hospital in the city of São Paulo between 2018 and 2022.

## METHODS

This study was approved by the Ethics in Research Committee (CAAE: 67959423.4.0000.5505; protocol: 6.090.376) and conducted as a retrospective and observational analysis. We collected data from medical records of patients admitted to the Burn Care Unit (BCU) of a university hospital in São Paulo between January 1, 2018 and December 31, 2022, using the Research Electronic Data Capture (REDCap) platform, a secure application for data collection and management.

### Inclusion and Exclusion Criteria

We included patients aged 18 years and older who were admitted to BCU during the study period and developed HAIs as defined by the HICC and the CDC criteria. 

We excluded individuals with incomplete or missing medical records, those who were discharged within 48 hours of admission, and patients who did not develop HAIs during hospital stay.

### Data Collection

We collected epidemiological and clinical data from medical records and stored them in a REDCap database. These data included demographic information (age, sex), pre-existing health conditions, percentage of body surface area burned (BSA), and the mechanism of injury. We also recorded additional data, such as length of hospital stay, laboratory and imaging test results, use of antimicrobials, and use of invasive devices. We analyzed culture samples to identify the etiologic agents of the infections and recorded patients’ mortality outcomes. We defined the patients’ sample after reviewing all the notification forms delivered by the HICC to the surveillance agencies.

### Identification of Healthcare-Associated Infections (HAIs)

HAIs were identified based on notifications from the HICC, following CDC criteria for diagnosing healthcare-associated infections. The most common HAIs evaluated included bloodstream infections, urinary tract infections, ventilator-associated pneumonia, and burn wound infections. Possible patient selection bias was reduced by reviewing all cases of HAI by two or more HICC members

### Statistical analysis

Statistical analyses were performed using the Statistical Package for the Social Sciences (SPSS) software, version 21.0. Results of continuous and discrete variables were evaluated for their distribution of normality with visual analysis and the Kolmogorov-Smirnov test. Continuous numerical variables were expressed as mean and standard deviation and median and interquartile range according to their distributions. Qualitative variables were expressed as numbers and percentages. We used the Student’s t-test to compare means of continuous variables with normal distribution, the Mann-Whitney for non-parametric continuous variables, and the Chi-square and Fisher’s exact tests to compare categorical variables.

## RESULTS

Based on the retrospective review of the medical records, 536 burn patients were consecutively admitted to the BCU of the Hospital São Paulo - Escola Paulista de Medicina - São Paulo Federal University, during the period from January 1, 2018, to December 31, 2022, and initially included in the study, all aged 18 and over. Within this group of patients, 88 patients (16%) developed HAIs, which the HICC consequently notified to the National Health Surveillance Agency (ANVISA).

In the period analyzed, 380 (70.8%) patients were male, and 156 (29.2%), female. The mean age was 41 years (range 18-88), 41 (range 18-88) in women and 39 (range 18-86) in men. Patients under 18 years of age who had not been hospitalized at the BCU for at least 24 hours were excluded from this study, as well as patients whose information in the medical records was incomplete. 

Among all patients hospitalized in the period, the mean burned body surface area (BSA) was 11.7% and the median was 7.4%, with a range of 0 to 70% for women (mean 12.5%) and 0 to 76% for men (mean 11.7%). All patients who were hospitalized without a burned body surface had electrical burns requiring monitoring or inhalation injury.

Unlike other samples, 87 (16.2%) patients were victims of electrical trauma; 122 had lesions caused by heated liquids; 182 lesions occurred due to flammable liquids; 13 injuries were from contact with chemicals; and 14 from contact with heated solids. Six patients had smoke inhalation injury alone.

Between 2018 and 2022, of the 536 patients, 25 patients died (4.5%), of whom 10 cases were distributed in 2018, four in 2019, four in 2020, six in 2021, and one case in 2022. The mean age of these patients was 75 years. The mean length of hospital stay was 21 days (range 1-174); 484 patients stayed for more than 72 hours in the unit. 

[Fig f1]

**Figure 1 f1:**
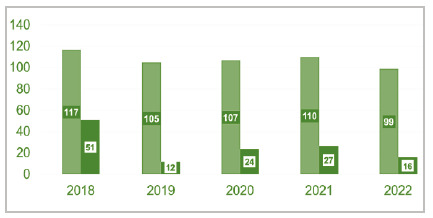
Distribution by year of hospitalizations in the burn care unit (ICU admissions, light green - n = 536) and cases of healthcare-associated infections (HAIs, dark green n = 88).

[Fig f2]

**Figure 2 f2:**
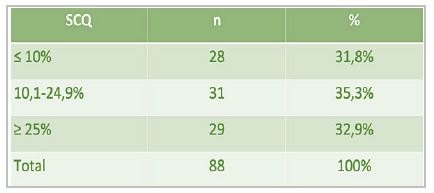
Distribution of total burned body surface area (BSA) among patients with HAI (n = 88).

Of the 88 patients with HAI, 68 were male (72%), with a mean age of 41 years (range 19-78) and a mean BSA of 20.4%, ranging from 1% (especially in patients who had isolated small skin burns associated with inhalation lesions, which occurred in 5.6% of the cases) to 70%.

In this series, burns were mainly caused by flammable liquids in 35 cases (39.7%), 22 (25%) by electrical trauma, and 13 (14.8%) by heated liquids. The mean length of hospital stay was 44.4 days. The overall mortality rate, considering the 88 patients sustaining HAI during hospitalization, was 16%, much higher than the mortality rate considering the group of all hospitalizations in the period (548 patients), which was 4.9% (27 cases).

During the five-year period, 130 HAIs were reported. Among the most frequent HAIs were Burned Area Infections (BAI - 67, 51.5%), followed by Bloodstream Infection (BSI - 20, 22.7%), Urinary Tract Infections Associated with the Indwelling Urinary Catheter (UTI-IUC - 17, 19.3%), and Ventilator-associated Pneumonia (VAP - 14, 15.9%). Other infections described and reported in smaller numbers were nosocomial pneumonia and tracheobronchitis (nine cases), osteomyelitis (three cases), and Clostridium spp. infection (one case).

[Fig f3]

**Figure 3 f3:**
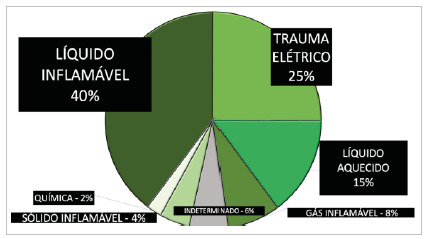
Mechanisms of burns distributed by agents (n = 88 patients).

[Fig f4]

**Figure 4 f4:**
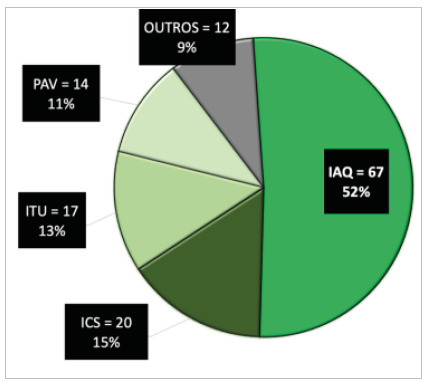
Topography of HAIs (n = 130; HAIs, 88 patients).

A total of 141 microorganisms were identified through culture, of which 101 (71.6%) were gram-negative bacteria, 30 (21.2%) were gram-positive, and 10 (7.1%) were fungi. In three cases, the microorganism was not identified. Of the 130 reported HAIs, 13 cases were caused by two microorganisms (10%), while in three there was no identified agent (2.3%). The remaining cases (115 - 87.8%) had a single agent identified. 

Considering all HAIs, the most frequent microbiological agents were Acinetobacter spp., in 34 cultures (24.1%), Pseudomonas spp., in 24 (17%), and Klebsiella spp., in 23 cultures (16.3%). As for gram-positive bacteria, there was a predominance of Enterococcus spp. (9.2%) and Staphylococcus aureus (7%).

[Fig f5]

**Figure 5 f5:**
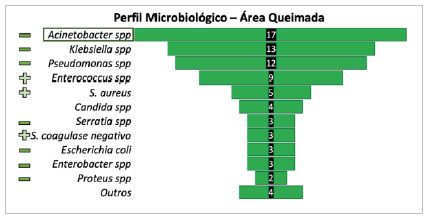
Most frequent microorganisms considering burned area infections (BAI - n = 67 cases, 78 microorganisms).

Considering each HAI topography, in this series there were 67 cases of BAI, with a predominance of gram-negative microorganisms, of which 17 were by Acinetobacter spp. (25.3%), 13 cases by Klebsiella spp. (19.4%), and 12 by Pseudomonas spp. (17.9%).

Regarding Bloodstream Infections, of the 20 reported cases, six were caused by Acinetobacter spp. (30%), four by Staphylococcus spp. (20%), three by Klebsiella spp. (15%), and three by Enterococcus spp. (15%).

As for UTI-IUC, of the 17 cases described, there was a predominance of Pseudomonas spp. (8 cases - 47%), followed by Acinetobacter spp. (three cases - 17.6%). Other agents were less frequent, such as Klebsiella spp. (two cases - 11.7%), Trichosporon spp. (two cases - 11.7%), and Enterococcus spp.

In the 14 VAP cases, 17 microorganisms were identified in culture, the most frequent being Acinetobacter spp. (eight - 57.1%), Klebsiella spp. (three - 21.4%), Pseudomonas spp. (two - 14.2%), and Staphylococcus spp. (two - 14.2%). 

[Fig f6]

**Figure 6 f6:**
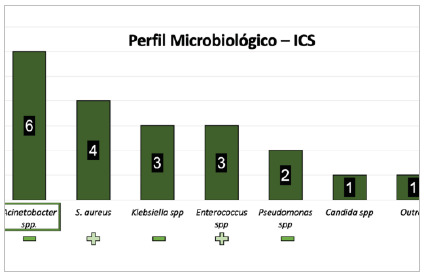
Most frequent microorganisms considering bloodstream infections (BSI - n = 20 infections).

[Fig f7]

**Figure 7 f7:**
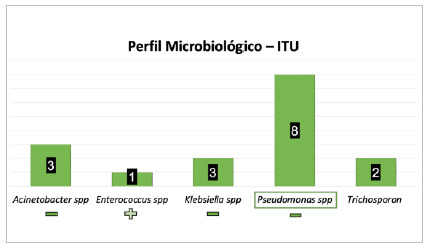
Most frequent microorganisms considering urinary tract infections (UTI - n = 17 infection).

[Fig f8]

**Figure 8 f8:**
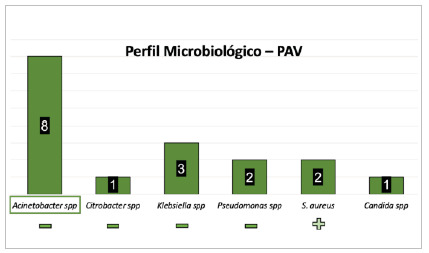
Most frequent microorganisms considering ventilator-associated pneumonia (VAP - n = 14 infections, 17 microorganisms).

Were submitted the variables burned body surface area (BSA), length of hospital stay, and Abbreviated Burn Severity Index (ABSI) score to the Shapiro-Wilk statistical normality test, with no normal distribution (BSA: W Statistic 0.902, p-value 6.14×10−6; ABSI: W Statistic 0.946, p-value 1.16×10−3; Days of hospitalization: W Statistic 0.870, p-value 2.90×10−7).

These variables were related to the occurrence of more than one infection during hospitalization. Of the 88 patients with HAIs, 22 (25%) had more than one infection episode. We correlated the variables BSA, length of hospital stay, and ABSI between the groups with one and more than one HAI during hospitalization. The Mann-Whitney U test showed statistical significance for the difference between these two groups in length of hospital stay (U Statistic 412.0, p-value 0.0025), BSA (U Statistic 503.5; p-value 0.0324), and ABSI (U Statistic 307.5, p-value 0.000043).

As for the types of infection, the Kruskal-Wallis test indicated no statistically significant differences between the types of nosocomial infection (BAI, UTI-IUC, VAP, and BSI) for the analyzed variables, suggesting that these types of infection have similar characteristics regarding length of hospital stay, BSA and ABSI score within the study sample.

## DISCUSSION

Most studies in literature present data on healthcare-associated infections (HAIs) grouped together with their microbiological profiles. This method of data presentation can hinder accurate evaluation and consequently discussion, since each infectious process has unique characteristics and distinct microbial profiles. By separating the data by type of infection, it is possible to infer more precise conclusions^7^ ^-^ ^9^. 

A study conducted by Appelgren et al. (2002) in Sweden evaluated 230 burn patients and observed that 36% of them developed infections, mainly of bloodstream origin (BSI). This contrasts with the present study, in which burned area infections (BAI) were the most frequent (51.5%), followed by BSI (13.8%). These differences may occur due to variations in management and care protocols between burn care units in different world regions^7^.

In Brazil, Santucci et al. (2003) conducted a study in a burn center for seven years, involving 320 patients, of whom 55% developed infections. In contrast to our study, they reported a predominance of gram-positive organisms at all infection sites, except for urinary tract infections (UTIs), which are strongly associated with the use of intravascular catheters. In our analysis, however, gram-negative bacteria were predominant (71.7% of all HAIs), with Acinetobacter spp. and Pseudomonas spp. being the most frequent pathogens. This shift in microbial prevalence may reflect changes in antibiotic use and resistance patterns over time or differences in hospital infection control practices^8^. 

Gragnani et al. (2014) conducted a study in the same burn of the present study, but over a period of ten years prior to ours. In their study, coagulase-negative Staphylococcus, Pseudomonas aeruginosa, and Acinetobacter baumannii were the most prevalent pathogens. Although our study also identified Pseudomonas spp. and Acinetobacter spp. as significant pathogens, we observed a clear shift towards a higher overall prevalence of gram-negative microorganisms. This may indicate evolving resistance patterns and the increasing role of gram-negative bacteria in burn wound infections over time^9^. 

Chen et al. (2019) published a retrospective study in Taiwan involving 709 patients, in which 11.3% developed HAIs. Similar to our findings, 50.7% of infections in their study were due to gram-negative bacteria. However, they reported a higher rate of fungal infections (21.2%), while fungi accounted for only 7.1% of infections in our series. This discrepancy may be attributed to differences in patient populations, hospital protocols, or environmental factors that influence fungal growth^10^. 

Escadón-Vargas et al. (2020) conducted a prospective study in Colombia involving 165 burn patients, with 27.9% developing HAIs. The predominant pathogens were Staphylococcus aureus, followed by Pseudomonas aeruginosa and Acinetobacter baumannii. In contrast, our study found a predominance of gram-negative bacteria, particularly Acinetobacter spp. (25.3%) and Klebsiella spp. (19.4%) in burn wound infections. While their sample had an average of 12% of burned BSA, our patients had a higher average of 20.4%, which may partially explain the higher proportion of gram-negative pathogens in our cohort, as more extensive burns may provide a more favorable environment for these bacteria^11^. 

Bourgi et al. (2020) retrospectively evaluated 475 patients admitted to a burn unit in Lebanon over five years. In their study, 54% of patients developed infections, in which gram-positive microorganisms, particularly Staphylococcus aureus, were the most common. This contrasts with our findings, where gram-negative bacteria were predominant. Differences in geographic regions, hospital protocols, and antibiotic stewardship programs may explain the variation in microbial prevalence among studies^12^. 

Choong et al. (2024) conducted a comprehensive review including 17 studies to assess the impact of infections in burn patients on length of stay (LS) and outcomes. Most studies found statistical significance in LS increase with the occurrence of HAIs, with 81% of studies showing a greater than 1.5-fold increase and 50% reporting a 2.0-fold increase. These results, according to our study, where the occurrence of HAI increased LS by 2.0 times (21 days for the general population vs. 42.5 days in patients who developed HAIs), increase morbidity, mortality, and costs^13^. 

Dey et al. (2023) retrospectively evaluated 100 patients for one year in a tertiary hospital in Bangladesh. Like other low-income countries, which generally have a higher incidence of HAI in burn patients, they described the occurrence of 42% of HAIs, including only skin burn infection and sepsis. In their series, Staphylococcus spp. was the main microorganism identified. These findings contrast with ours, in which HAI occur in 16% of patients and gram-negative microorganisms were predominant^14^. 

Gueno Risseto et al. (2024) conducted a retrospective study comparing the occurrence of HAI in a burn unit in Brazil in two different periods, 2015 and 2020. They did not identify changes in the microbiological profile over time, with Pseudomonas aeruginosa being more frequent in both periods, followed by Acinetobacter baumannii and Staphylococcus aureus. This contrasts with our study, which observed a change in the microbiological profile and the predominance of Acinetobacter baumannii^15^. 

Öncül et al. (2014) retrospectively described a cohort with sociodemographic characteristics similar to our sample. In their series, they found the same pattern of microbiological changes over time, with a decrease in gram-positive infections and an increase in gram-negative ones^16^. 

Another interesting study was conducted by Cato et al. (2023) in Birmingham, UK, assessing the change in microbiological profile after 14 years in the same burn unit. Similar to our findings, over time there was a greater participation of Acinetobacter baumannii in colonization and in wound infections, with a progression of fungal infections^17^. 

Our research’s results demonstrate a clear change in the predominance of gram-negative bacteria in HAIs among burn patients, particularly Acinetobacter spp. and Pseudomonas spp. This shift highlights the importance of continued surveillance of microbial profiles and the need for targeted antibiotic therapies that prioritize coverage for gram-negative organisms.

This study is limited by its retrospective design, which may result in incomplete or missing data. In addition, it was conducted in a single burn unit, which may limit the generalizability of the findings to other contexts. Variations in infection control practices, antimicrobial use, and patient demographics may also influence observed trends in microbial prevalence. The generalization of inferences about the collected data is limited. Moreover, it was non-randomized, which may bring potential bias with confounding variables.

The high frequency of Healthcare-Associated Infections (HAIs) among our patients has a potential impact on morbidity and mortality rates. The determination of their microbiological profile is relevant and can be used to compare the evolution over time and with other series. This can improve outcomes in burn patients, together with the adoption of antimicrobial stewardship programs, strict infection control, and surveillance policies.

Despite these limitations, this study highlights the evolving microbial landscape in burn care, particularly the increasing prevalence of gram-negative pathogens. These findings have important implications for infection control and treatment strategies in burn units. The results emphasize the need for continued surveillance of antimicrobial resistance patterns.

## CONCLUSION

Unlike other findings in literature, gram-negative bacteria are the predominant cause of healthcare-associated infections in burn patients at our institution. Acinetobacter spp. infections were particularly prevalent. The findings highlight the importance of adapted infection control measures and the need for empirical antibiotic regimens that cover gram-negative bacteria to reduce morbidity and mortality in this vulnerable population.
